# *Lactobacillus plantarum* 17–5 attenuates *Escherichia coli*-induced inflammatory responses via inhibiting the activation of the NF-κB and MAPK signalling pathways in bovine mammary epithelial cells

**DOI:** 10.1186/s12917-022-03355-9

**Published:** 2022-06-28

**Authors:** Ke Li, Ming Yang, Mengyue Tian, Li Jia, Jinliang Du, Yinghao Wu, Lianmin Li, Lining Yuan, Yuzhong Ma

**Affiliations:** 1 College of Veterinary Medicine, Hebei Agricultural University, Baoding, 071001 Hebei China; 2 College of Life Science and Food Engineering, Hebei University of Engineering, Handan, 056038 Hebei China; 3 Key Laboratory of Freshwater Fisheries and Germplasm Resources Utilization, Ministry of Agriculture, Freshwater Fisheries Research Center, Chinese Academy of Fishery Sciences, Wuxi, 214081 China

**Keywords:** *Lactobacillus plantarum*, *Escherichia coli*, Inflammation, NF-κB, MAPK, Bovine mammary epithelial cell

## Abstract

**Background:**

Mastitis is one of the most prevalent diseases and causes considerable economic losses in the dairy farming sector and dairy industry. Presently, antibiotic treatment is still the main method to control this disease, but it also brings bacterial resistance and drug residue problems. *Lactobacillus plantarum* (*L. plantarum*) is a multifunctional probiotic that exists widely in nature. Due to its anti-inflammatory potential, *L. plantarum* has recently been widely researched in complementary therapies for various inflammatory diseases. In this study, the apoptotic ratio, the expression levels of various inflammatory mediators and key signalling pathway proteins in *Escherichia coli*-induced bovine mammary epithelial cells (BMECs) under different doses of *L. plantarum* 17–5 intervention were evaluated.

**Results:**

The data showed that *L. plantarum* 17–5 reduced the apoptotic ratio, downregulated the mRNA expression levels of *TLR2*, *TLR4*, *MyD88*, *IL1β*, *IL6*, *IL8*, *TNFα*, *COX2*, *iNOS*, *CXCL2* and *CXCL10*, and inhibited the activation of the NF-κB and MAPK signalling pathways by suppressing the phosphorylation levels of p65, IκBα, p38, ERK and JNK.

**Conclusions:**

The results proved that *L. plantarum* 17–5 exerted alleviative effects in *Escherichia coli*-induced inflammatory responses of BMECs.

**Supplementary Information:**

The online version contains supplementary material available at 10.1186/s12917-022-03355-9.

## Background

Mastitis is one of the most prevalent diseases in dairy cows, causing severe economic losses and restricting the development of the dairy cow industry [1]. It is usually caused by infection of the mammary gland with pathogens, such as *Escherichia coli*, one of the major environmental pathogens responsible for dairy cow mastitis [2, 3]. Coliform mastitis is normally characterized by severe local inflammatory responses and systemic symptoms, and can even cause death under the most serious circumstances [4]. Therefore, it is essential to determine the primary pathogens and inhibit inflammatory reactions to control this disease.

Currently, there is no exact and effective treatment for dairy cow mastitis in the clinic, and antibiotics are often used to control the progression of disease. Despite the positive results of this treatment approach, the excessive use of antibiotics also brings drug resistance and residue problems [5, 6]. Considering these potential issues, many countries, such as China and the European Union, have already restricted antibiotics in animal feed [7]. Hence, finding alternative safe and effective drugs is of great importance for human health and animal welfare.

*L. plantarum* is a versatile and abundant probiotic found in diverse environments ranging from food to animal and human gastrointestinal tracts [8]. *L. plantarum* is widely used for food processing and livestock feed due to its potential health benefits and biosafety [9]. In recent years, with the deepening of research, the potential anti-inflammatory properties of *L. plantarum* have come into the sight of researchers. Yue et al. found that *L. plantarum* could reduce the expression of *TLR4*, *IL6*, and *TNFα* as well as jejunal injury and had a protective effect on diarrhoea caused by enterotoxigenic *Escherichia coli* [10]. Tian et al.’s research showed that oral supplementation with *L. plantarum* TW1–1 decreased inflammation and modulated gut microbiota in DEHP-induced testicular damage mice [11]. Frola et al. pointed out that *L. plantarum* CRL 1716 provided good therapeutic effects on dairy cow mastitis after intramammary inoculation in lactating cows [12]. Thus, we hypothesized that *L. plantarum* might have a protective effect against inflammatory injury in mastitis cows. However, our analysis of the present literature reveals that related basic research is still limited.

In this study, the probiotic *L. plantarum* 17–5 with possible anti-inflammatory activity was selected. This study aimed to investigate the potential protective effects of this probiotic on *Escherichia coli*-induced inflammatory responses of BMECs and lay an excellent foundation for developing relevant microecological preparations.

## Results

### Dose effect of *L. plantarum* 17–5 on cell viability

**Fig. 1:**
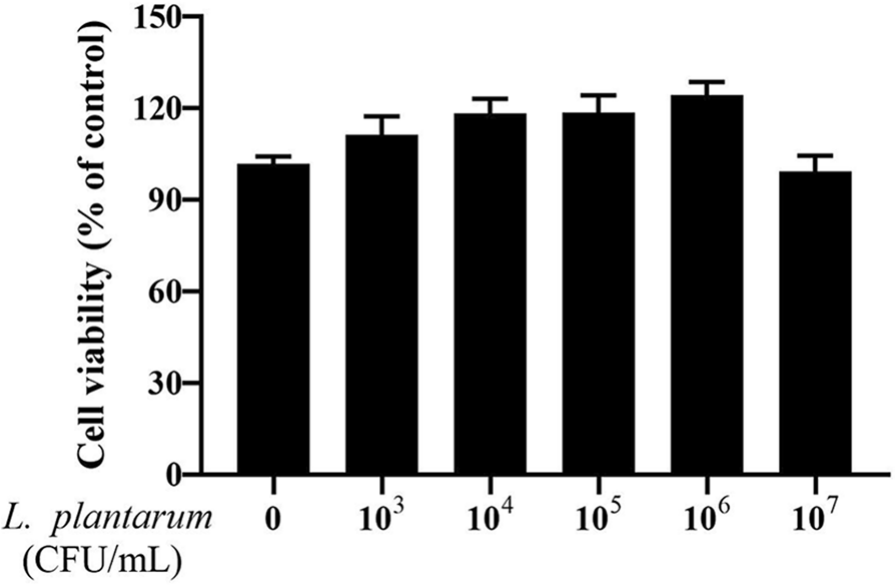
The cytotoxic effects of *L. plantarum* 17–5 on BMECs. Cell viability was assessed at gradient concentrations of *L. plantarum* 17–5 (0, 10^3^, 10^4^, 10^5^, 10^6^ and 10^7^ CFU/mL) for 3 h by CCK-8 assay. The data were presented as the mean ± SEM, *n* = 5

To evaluate the toxicity of *L. plantarum* 17–5 on BMECs, the CCK-8 assay was performed to detect cell viability. As shown in Fig. 1, none of the tested concentrations showed cytotoxicity on BMECs, except the 10^7^ CFU/mL dose. Therefore, three concentrations (10^4^, 10^5^ and 10^6^ CFU/mL) were selected for subsequent experiments.

### Effect of *L. plantarum* 17–5 on apoptosis of BMECs

**Fig. 2:**
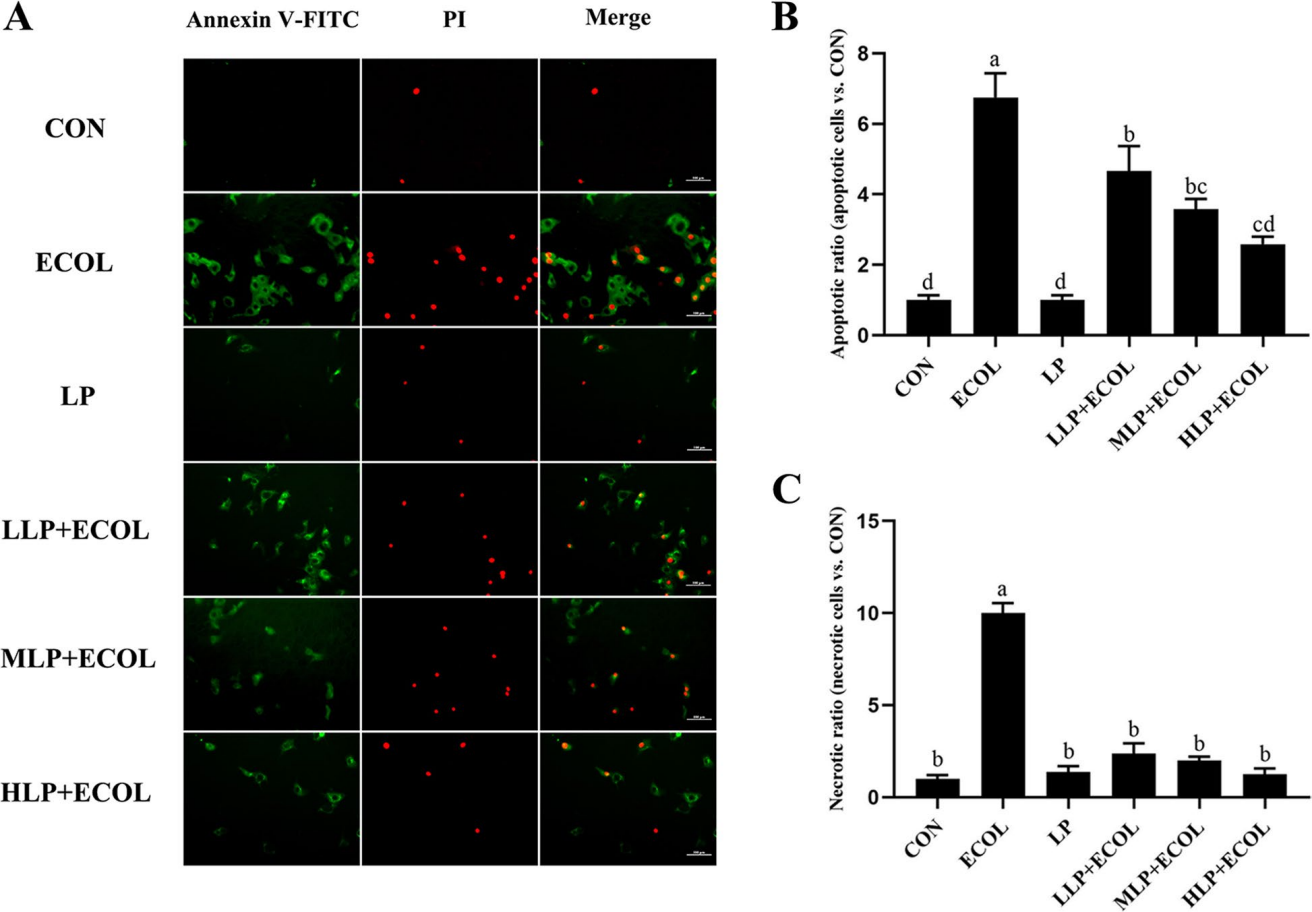
**A** Apoptosis was determined by Annexin V-FITC/PI staining. The apoptotic cells showed green fluorescence (FITC-positive and PI-negative), necrotic cells showed both green and red fluorescence (FITC-positive and PI-positive), and normal cells had no fluorescence signal (FITC-negative and PI-negative). Scale bars: 100 μm. (**B, C**) The apoptotic ratio and necrotic ratio in each group. Values from five visual fields were shown as mean ± SEM. The same letter on top of the bars indicated no significant difference, however, different letters indicated significant difference (*P* < 0.05). The same as the following figures

The effects of varying *L. plantarum* 17–5 doses on the apoptosis of BMECs were analysed (Fig. 2A). The results showed that the apoptotic ratio in the ECOL group increased significantly (*P* < 0.05) compared with those in the CON group and decreased significantly (*P* < 0.05) in the *L. plantarum* 17–5 preconditioning group compared with those in the ECOL group (Fig. 2B). Similar results were seen in the necrotic ratio. The necrotic ratio in each dose preconditioning group decreased significantly (*P* < 0.05) compared with those in the ECOL group (Fig. 2C).

### Effect of *L. plantarum* 17–5 on the mRNA expressions of TLRs and MyD88

**Fig. 3:**
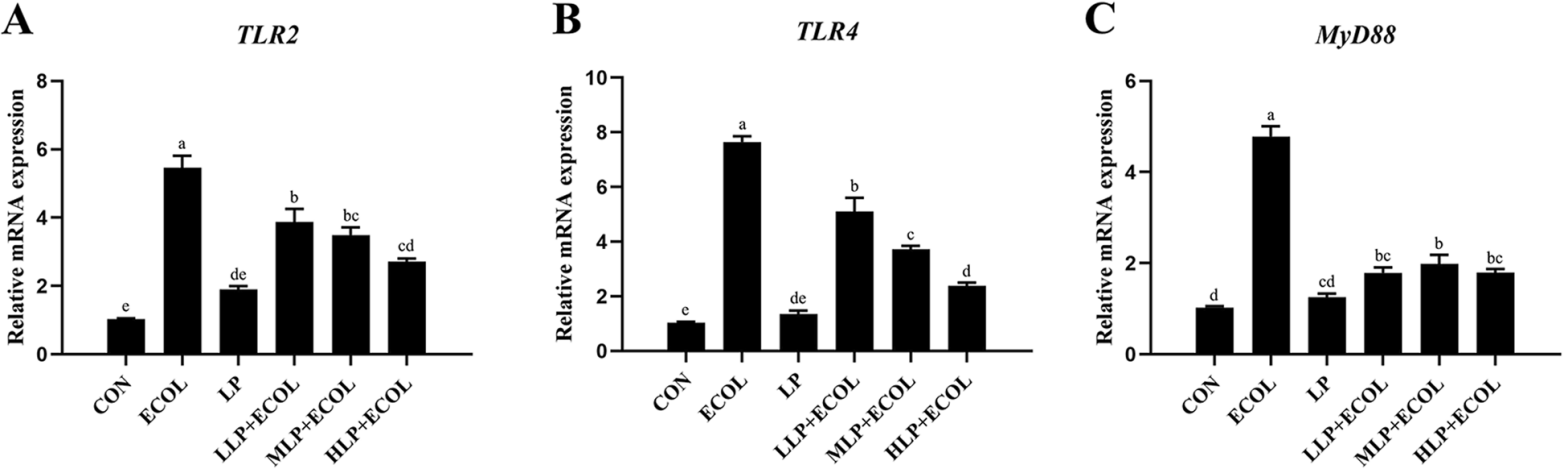
Effects of *L. plantarum* 17–5 on TLRs and MyD88 mRNA expression in *E. coli*-treated BMECs. The mRNA expression levels of *TLR2*, *TLR4* and *MyD88* (**A-C**) were evaluated with qRT–PCR in BEECs. The values were presented as the means ± SEM of three independent experiments

The expression levels of *TLR2*, *TLR4* and *MyD88* were measured by real-time PCR. As shown in Fig. 3, after *E. coli* induction, the expression levels of *TLR2*, *TLR4* and *MyD88* mRNA increased compared with those in the CON group (*P* < 0.05). Pretreatment with three different doses of *L. plantarum* 17–5 significantly reduced the expression of *TLR2*, *TLR4* and *MyD88* mRNA after *E. coli* infection (*P* < 0.05) (Fig. 3A-C).

### Effect of *L. plantarum* 17–5 on the mRNA expression of inflammatory mediators and chemokines

**Fig. 4:**
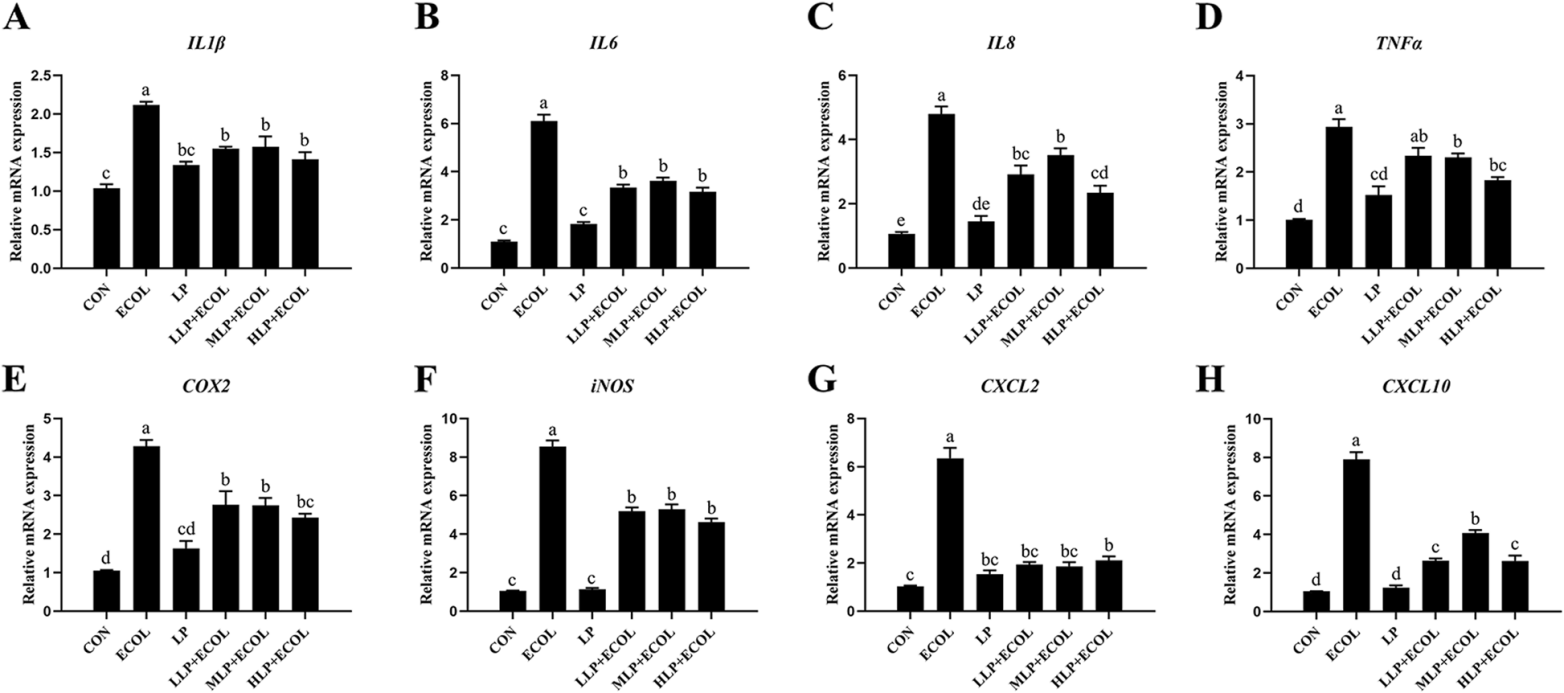
Effects of *L. plantarum* 17–5 on the inflammatory mediator and chemokine mRNA expression in *E. coli*-treated BMECs. The mRNA expression levels of *IL1β IL6*, *IL8*, *TNFα*, *COX2*, *iNOS*, *CXCL2* and *CXCL10* (A-H) in BEECs were detected with the same methods as above. Values were presented as the means ± SEM from three independent experiments

The expression levels of *IL1β*, *IL6*, *IL8*, *TNFα*, *COX2*, *iNOS*, *CXCL2* and *CXCL10* were examined using the same method as above. The results showed that *E. coli* increased the mRNA levels of all the indicators (*P* < 0.05). However, these increases were significantly mitigated by pretreatment of *L. plantarum* 17–5 (*P* < 0.05) (Fig. 4A-H).

### Effect of *L. plantarum* 17–5 on the protein expression of the NF-κB and MAPK pathways

**Fig. 5:**
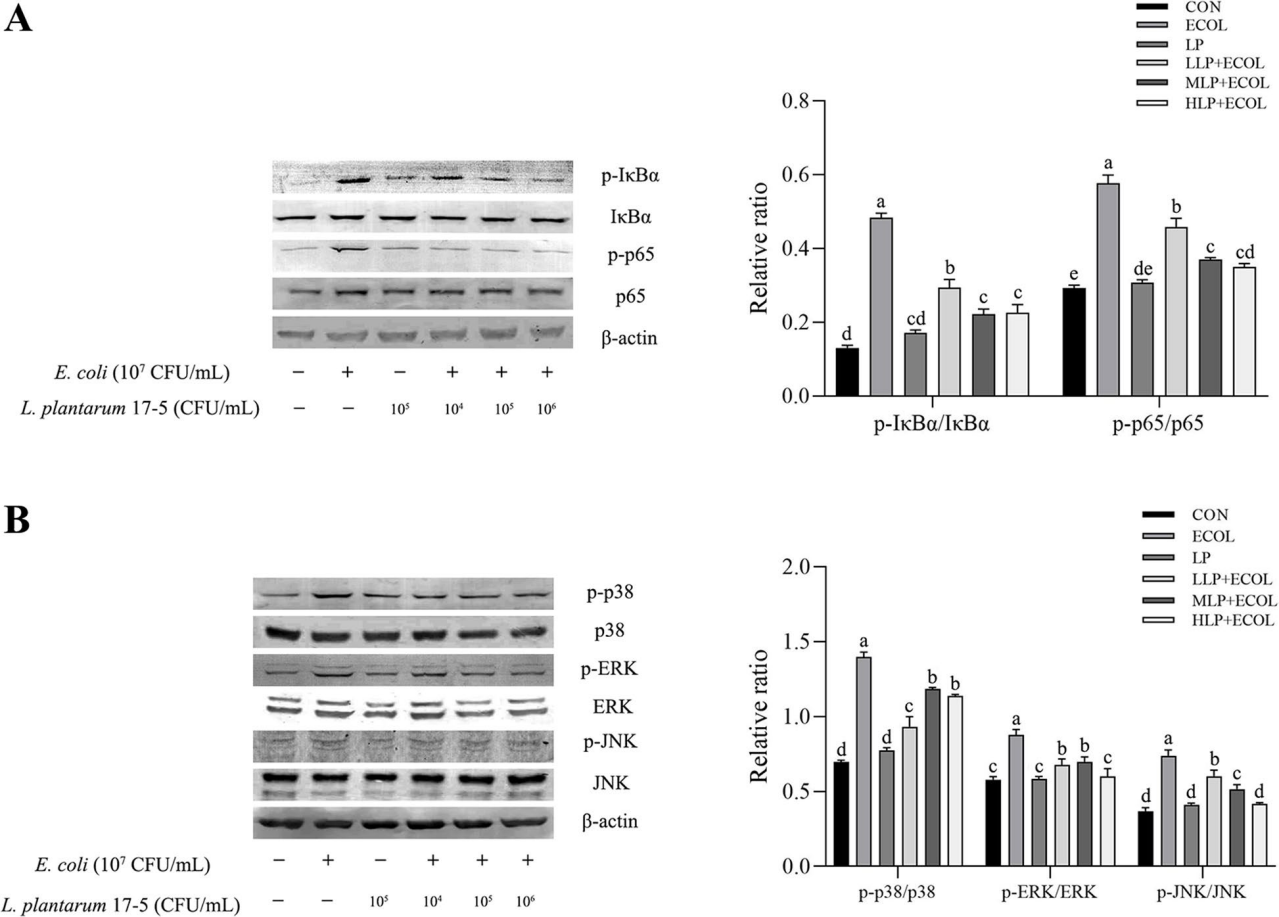
Inhibitory effects of *L. plantarum* 17–5 on NF-κB (**A**) and MAPK (**B**) phosphorylation in BMECs. Data were represented as the mean ± SEM of three independent experiments

Western blot analysis demonstrated that *E. coli* upregulated the phosphorylation levels of p65, IκBα, p38, ERK and JNK (*P* < 0.05). However, these upregulations were inhibited by pretreatment of *L. plantarum* 17–5 (Fig. 5A, B).

## Discussion

Due to the multiple problems caused by the overuse of antibiotics, several alternative biologics, including beneficial microbes, are being considered to treat and prevent dairy cow mastitis [13]. Several studies suggested that some lactic acid bacteria showed promising effects in treating bovine mastitis [14, 15], among which *L. plantarum* is a typical Lactobacillus species with various beneficial effects on host metabolic health. Martín et al. successfully isolated *Lactobacillus fermentum*, *Lactobacillus aerogenes* and *Lactobacillus salivarius* from the milk of healthy females, thereby confirming the existence of probiotics in normal healthy mammary gland tissue [16], this study provides a basis for probiotic treatment in humans. However, there are still some potential problems with this biological therapy, especially when it acts directly on the mammary gland tissue. A recent study reported that intramammary injections of *Lactococcus lactis* (approximately 10^7^ CFU) might elicit a suppurative inflammatory response in the murine model [17]. In light of these challenges, we used a lower dose and treatment time than the above report. Meanwhile, the cytotoxicity assay showed that test doses of *L. plantarum * 17-5 were not toxic to BMECs. Given this result and subsequent indicators, we believe that the test conditions were safe and effective for the cell.

Identifying pathogens is the first step in defence against invading pathogens in the immune system [18]. Mammary epithelial cells have many pattern recognition receptors (PRRs) that are distributed on the cell surface or in the cytoplasm, such as toll-like receptors (TLRs) [19]. Some TLRs, which span the cell membrane, can recognize pathogen-associated molecular patterns (PAMPs). For example, TLR2 recognizes bacterial lipoproteins, and TLR4 recognizes exogenous ligands such as LPS [20]. *Escherichia coli* is an important causative agent of dairy cow mastitis due to its higher incidence rate than other pathogenic microbes [21]. After *E. coli* invades cow mammary tissue, the TLR2 and TLR4 receptors are activated, which mostly leads to MyD88 downstream signalling and consequently activates a series of downstream pathways, kinases and transcription factors [18, 22]. In this study, we found that the mRNA expression levels of *TLR2*, *TLR4* and *MyD88* were upregulated after incubation with *E. coli* at 8 h, whereas preincubation with *L. plantarum* 17–5 significantly suppressed these changes. Based on these results, we suspect that *L. plantarum* 17–5 has regulatory effects on downstream inflammatory genes and pathways.

Bacterial infections are usually accompanied by severe inflammatory responses [23]. Excessive inflammatory mediator production can promote inflammatory injury and induce cell apoptosis [24, 25]. TNFα is an inflammatory mediator that plays a proinflammatory role in early inflammation [26]. IL1β and IL6 participate in the occurrence and development of inflammation by activating the expression of other proinflammatory cytokines and modulating chemokine expression [27]. PGE2 and NO were proven to induce intense inflammation with trace amounts in previous research, whereas COX2 and iNOS are key enzymes in their biosynthesis, respectively [28]. Furthermore, some chemokines, such as IL8, CXCL2 and CXCL10, have also been implicated in inflammatory injury [29]. Previous studies have shown that *E. coli* can stimulate host cells to release various proinflammatory mediators, including IL1β, IL6, TNFα and NO, while inducing cell apoptosis [30, 31]. Our results showed that pretreatment with different doses of *L. plantarum* 17–5 reduced the expression of *IL1β*, *IL6*, *IL8*, *TNFα*, *COX2*, *iNOS*, *CXCL2* and *CXCL10* during *E. coli* infection. We also evaluated the effect of *L. plantarum* 17–5 on *E. coli*-induced apoptosis. As expected, *L. plantarum* 17–5 inhibited the apoptosis of these cells. This result is consistent with a previous report that probiotics can inhibit induced apoptosis [31].

The MAPK and NF-κB signalling pathways, which are downstream of TLRs, play a key role in regulating cellular proliferation, apoptosis, and inflammation [32]. NF-κB is a pleiotropic transcription factor involved in the control of proinflammatory gene expression, such as *TNFα*, *IL6*, *COX2* and *iNOS* [33]. In the quiescent state, NF-κB, as an inactive complex, binds to IκB, an NF-κB inhibitor. Under the action of upstream factors, IκB is phosphorylated and dissociates from NF-κB, allowing NF-κB p65 to translocate to the nucleus and thus activate related gene transcription [34]. The MAPK pathways include p38 MAPK, ERK1/2, and JNK, and this pathway controls the synthesis and release of cytokines during the inflammatory response [35]. The activation of each MAPK depends on multiple upstream kinases with unique cascade reactions [36]. A previous report indicated that *Lactobacillus plantarum* could inhibit the inflammatory response by modulating the NF-κB and MAPK pathways [37, 38]. Similarly, our data suggest that *L. plantarum* 17–5 markedly decreased the phosphorylation of key proteins in the NF-κB and MAPK signalling pathways.

## Conclusions

In conclusion, *L. plantarum* 17–5 can attenuate *E. coli*-induced inflammatory responses by inhibiting the mRNA expression of inflammatory mediators and the activation of the NF-κB and MAPK signalling pathways in BMECs and may be a potential therapeutic agent for dairy cow mastitis. However, due to the limitations of the in vitro model, the biological significance of these findings needs further investigation in vivo.

## Materials and methods

### Chemicals and reagents

Dulbecco’s modified Eagle’s medium/Ham’s F-12 nutrient mixture (DMEM/F12) and foetal bovine serum (FBS) were purchased from Gibco (Grand Island, NY), hydrocortisone from Sigma–Aldrich (MO, USA), de Man Rogosa Sharpe (MRS) broth and Luria-Bertani (LB) broth from Aobox (Beijing, China), cell counting kit-8 (CCK-8), RIPA lysis buffer, BCA protein assay kit and BCIP/NBT colour development kit from Solarbio (Beijing, China), and ultrapure RNA extraction kit from CWBIO (Beijing, China). Uelris Il RT–PCR System for First-Strand cDNA Synthesis and AugeGreen™ qPCR Master Mix from US Everbright Inc. (CA, USA), and an Annexin V-FITC Apoptosis Detection Kit from Beyotime (Shanghai, China). Primary antibodies against p38 (Catalog #8690 T), phospho-p38 (Catalog #4511 T), ERK (Catalog #4695 T), phospho-ERK (Catalog #4370 T), JNK (Catalog #9252 T), phospho-JNK (Catalog #4668 T) and IκBα (Catalog #4812S) were acquired from Cell Signaling Technology (Danvers, MA, USA), and antibodies against NF-κB p65 (Catalog #bs-0465R), NF-κB phospho-p65 (Catalog #bs-0982R), phospho-IκBα (Catalog #bsm-52169R) and β-actin (Catalog #bs-0061R) were purchased from Bioss (Woburn, MA, USA).

### Culture of bacterial strains and cells

*Lactobacillus plantarum* 17–5 (ATCC 8014, American Type Culture Collection, Manassas, VA, USA) was cultured by static culture with MRS broth at 37 °C for 24 h. *Escherichia coli* O111:K58 (B4) (ATCC 43887) was grown in LB broth at 37 °C for 12 h with shaking. After three generations, bacterial strains at the logarithmic growth phase were used for subsequent experiments. Primary cultures of bovine mammary epithelial cells (BMECs) from the mammary glands of Holstein dairy cows by modification of previously reported protocol [39], these changes were made to make cells grow better. The acquired mammary tissues were washed using PBS with constant stirring to remove residual milk, finely sliced into small pieces (0.5 cm^3^) and cultured in humidified air containing 5% CO_2_ at 37 °C. When the cells were 60–80% confluent, the tissue was removed. The fibroblasts were removed and subsequent epithelial cells were enriched according to their different sensitivity to 0.25% trypsin-EDTA. Next, the cells were inoculated into new flasks and cultured in DMEM/F12 supplemented with 15% FBS, 0.1% hydrocortisone, 0.025 M HEPES, 100 U/mL penicillin–streptomycin, and maintained in the same culture conditions as described above.

### Cell viability assay

The *L. plantarum* 17–5 cytotoxicity to BMECs was evaluated using the CCK-8 assay according to the manufacturer’s instructions. BMECs were seeded into 96-well microplates at a concentration of 1 × 10^4^ cells/well and cultured to 80–90% confluence. Cells were treated with varying concentrations of *L. plantarum* 17–5 (10^3^ to 10^7^ CFU/mL) for 3 h. Then, 10 μL CCK-8 solution was added to each well and further incubated for 2 h at 37°C. Subsequently, the absorbance at 450 nm was measured using a microplate reader.

### Cell immunofluorescence assay

BMECs were seeded in 96-well plates and cultured to 70–80% confluence as described above. The confluent cells were then treated as follows: CON group, DMEM/F12 alone; ECOL group, *E. coli* (10^7^ CFU/mL) infection alone; LP group, *L. plantarum* 17–5 (10^5^ CFU/mL) incubation alone for 3 h; (L, M, H) LP + ECOL group, *L. plantarum* 17–5 (10^4^, 10^5^ and 10^6^ CFU/mL) preincubation for 3 h before the addition of *E. coli* (10^7^ CFU/mL). After *E. coli* infection for 8 h, the medium was discarded, and the cells were washed with PBS. Subsequently, the cells were stained with an Annexin V-FITC / Propidium Iodide (PI) Apoptosis Detection Kit and observed under a fluorescence microscope. Five visual fields were randomly selected for microscopic observation and the positive cells were counted by ImageJ software (NIH, Bethesda, USA). The apoptotic ratio was calculated as the ratio between the number of apoptotic cells in the treatment group and the untreated control group. The same was done for the necrotic ratio.

### qRT–PCR analysis

Total RNA from BMECs was extracted using the Ultrapure RNA extraction kit according to the manufacturer’s instructions. The RNA integrity was assessed by agarose gel electrophoresis and the concentration and purity (the ratio of the OD_260_/OD_280_ and OD_260_/OD_230_) were measured on the Nanodrop 2000 (Thermo Fisher Scientific, Wilmington, DE, USA). Then the above RNA is reversely transcribed into cDNA by a reverse transcription kit for quantitative real-time PCR (qRT–PCR) analyses. The reaction procedures were as follows: 300 s at 95 °C followed by 40 cycles of 5 s at 95 °C, 30 s at 60 °C and 15 s at 72 °C. The PCR amplification efficiency was evaluated using standard curve analysis. The level of target gene expression was normalized to the *GAPDH* reference gene (For stability of the reference genes, please refer to supplementary material) and calculated using the 2^−ΔΔCt^ method, and the primer sequences are listed in Table 1.

**Table 1:** Sequences of primer used for qRT-PCR

Gene	Primer sequence (5′-3′)	Product sizes (bp)	GenBank accession no.
*TLR2*	CATTCCCTGGCAAGTGGATTATC	201	AY634629
	GGAATGGCCTTCTTGTCAATGG		
*TLR4*	AGCTTCAACCGTATCATGGCCTCT	166	NM_174198.6
	ACTAAGCACTGGCATGTCCTCCAT		
*MyD88*	AAGTACAAGCCAATGAAGAAAGAG	102	NM_001014382.2
	GAGGCGAGTCCAGAACCAG		
*IL-1β*	CCTCGGTTCCATGGGAGATG	119	NM_174093.1
	AGGCACTGTTCCTCAGCTTC		
*IL-6*	TGAAAGCAGCAAGGAGACACT	90	NM_173923.2
	TGATTGAACCCAGATTGGAAGC		
*TNF-α*	GGGCTTTACCTCATCTACTCACAG	132	NM_173966.3
	GATGGCAGACAGGATGTTGACC		
*IL-8*	ACACATTCCACACCTTTCCAC	149	AF232704
	ACCTTCTGCACCCACTTTTC		
*COX-2*	GGTGCCTGGTCTGATGATGT	124	NM_174445.2
	GATTAGCCTGCTTGTCTGGA		
*iNOS*	TGTCAGCGGCAAGCACCACATT	289	NM_001076799.1
	CGGCTGGTTGCATGGGAAAACT		
*CXCL-2*	ACCGAAGTCATAGCCACTCTC	218	NM_174299.3
	TCCAGATGGCCTTAGGAGGT		
*CXCL-10*	CTCGAACACGGAAAGAGGCA	117	NM_001046551
	TCCACGGACAATTAGGGCTT		
*GAPDH*	CACCCTCAAGATTGTCAGCA	103	NM_001034034.2
	GGTCATAAGTCCCTCCACGA		

### Western blot analysis

Proteins from cells were extracted using RIPA lysis buffer and quantified using a BCA protein assay kit. Protein samples (20 μg) were separated on 12% polyacrylamide-SDS gels, and resolved proteins were then transferred onto nitrocellulose membranes. The membrane was blocked with 5% skimmed milk for 1 h at room temperature. After incubation with primary and secondary antibodies, immunoblot signals were visualized with an NBT/BCIP colour development kit. The densities of the protein bands from three separate experiments were quantified by ImageJ software.

### Statistical analysis

All data are presented as the mean ± SEM from at least three independent experiments. Comparisons between the groups were evaluated by one-way ANOVA test with Tukey’s multiple comparisons test. *P* values < 0.05 were considered significantly different.

## Supplementary Information

**Additional file 1.** The original, full length blots of western blot.

**Additional file 2.** Analysis of reference genes expression stability.

## Data Availability

The original sequences we used for primer sequence design can be found in GenBank (https://www.ncbi.nlm.nih.gov/genbank/) under the accession numbers AY634629, NM_174198.6, NM_001014382.2, NM_174093.1, NM_173923.2, NM_173966.3, AF232704, NM_174445.2, NM_001076799.1, NM_174299.3, NM_001046551, and NM_001034034.2. The original, full-length western blot blots and analysis of reference genes expression stability are listed in the supplementary information (Additional files 1 and 2). Data generated during the presented study are available from the corresponding author (YZM) upon reasonable request.

## Abbreviations

BMECs: Bovine mammary epithelial cells
DEHP: Diethylhexylphthalate
TLRs: Toll-like receptors
PAMPs: Pathogen-associated molecular patterns
MyD88: Myeloid differentiation Factor 88
FITC: Fluorescein isothiocyanate
