# Supplementary material for: Lactobacillus plantarum 17–5 attenuates Escherichia coli-induced inflammatory responses via inhibiting the activation of the NF-κB and MAPK signalling pathways in bovine mammary epithelial cells
Source: BMC Vet Res. 2022 Jun 28;18:250. doi: 10.1186/s12917-022-03355-9 (PMC9238091; doi:10.1186/s12917-022-03355-9)
Supplement: Supplementary file 1 — Additional file 1. The original, full length blots of western blot. [file 12917_2022_3355_MOESM1_ESM.docx]

Additional file 1. The original, full length blots of western blot.

45 kDa


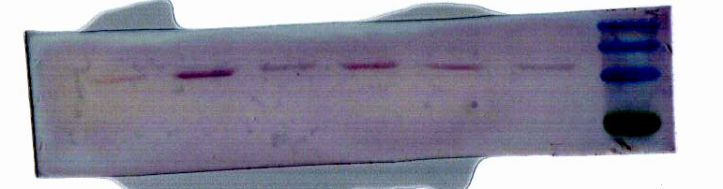


**p-IĸBα**

*E. coli* (10^7^ CFU/mL) - + - + + +

*L. plantarum* 17-5 (CFU/mL) - - 10^5^ 10^4^ 10^5^ 10^6^

36 kDa

60 kDa

35 kDa

25 kDa


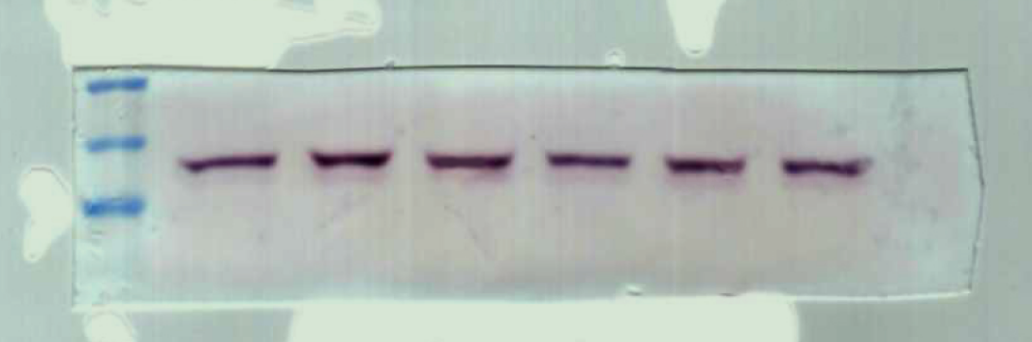


**IĸBα**

*E. coli* (10^7^ CFU/mL) - + - + + +

*L. plantarum* 17-5 (CFU/mL) - - 10^5^ 10^4^ 10^5^ 10^6^

60 kDa

45 kDa

35 kDa

36 kDa

80 kDa

60 kDa

45 kDa


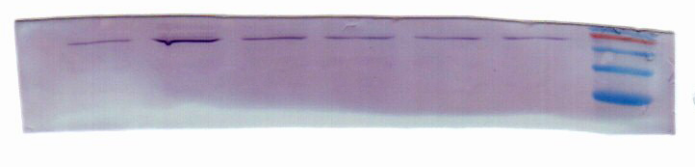


**p-p65**

*E. coli* (10^7^ CFU/mL) - + - + + +

*L. plantarum* 17-5 (CFU/mL) - - 10^5^ 10^4^ 10^5^ 10^6^

61 kDa


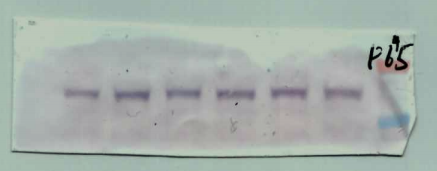


**p65**

*E. coli* (10^7^ CFU/mL) - + - + + +

*L. plantarum* 17-5 (CFU/mL) - - 10^5^ 10^4^ 10^5^ 10^6^

61 kDa

80 kDa

60 kDa


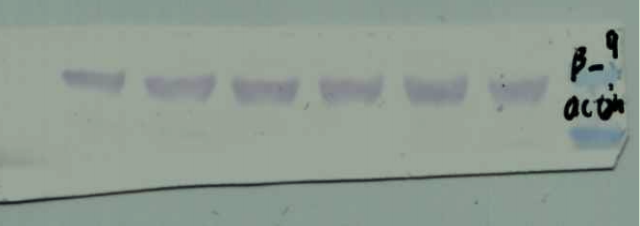


**β-actin**

*E. coli* (10^7^ CFU/mL) - + - + + +

*L. plantarum* 17-5 (CFU/mL) - - 10^5^ 10^4^ 10^5^ 10^6^

42 kDa

45 kDa

35 kDa


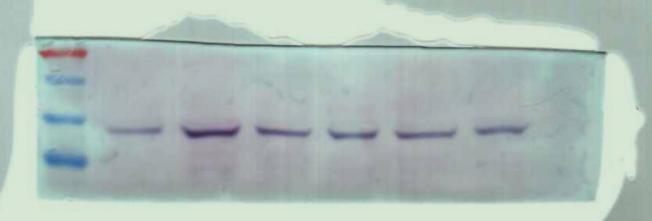


**p-p38**

*E. coli* (10^7^ CFU/mL) - + - + + +

*L. plantarum* 17-5 (CFU/mL) - - 10^5^ 10^4^ 10^5^ 10^6^

60 kDa

43 kDa

45 kDa

35 kDa


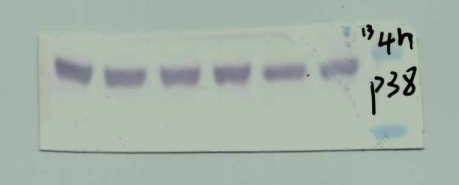


**p38**

*E. coli* (10^7^ CFU/mL) - + - + + +

*L. plantarum* 17-5 (CFU/mL) - - 10^5^ 10^4^ 10^5^ 10^6^

40 kDa

45 kDa

35 kDa

60 kDa


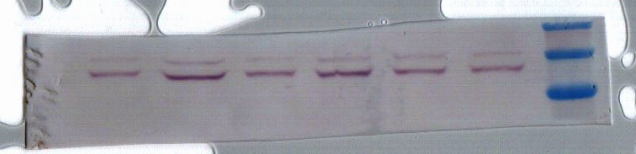


**p-ERK**

*E. coli* (10^7^ CFU/mL) - + - + + +

*L. plantarum* 17-5 (CFU/mL) - - 10^5^ 10^4^ 10^5^ 10^6^

44 kDa

45 kDa

42 kDa

35 kDa


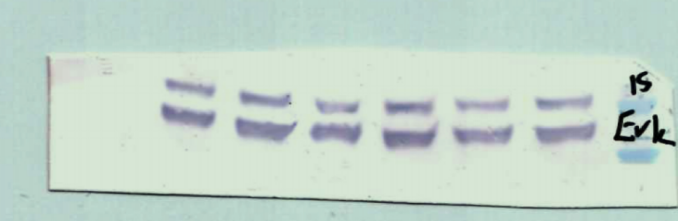


**ERK**

*E. coli* (10^7^ CFU/mL) - + - + + +

*L. plantarum* 17-5 (CFU/mL) - - 10^5^ 10^4^ 10^5^ 10^6^

44 kDa

45 kDa

42 kDa

35 kDa


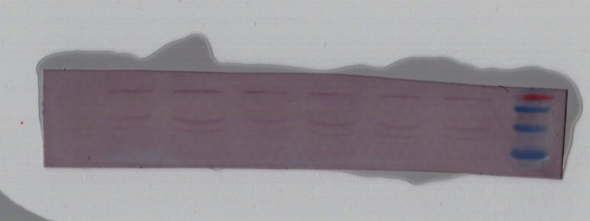


**P-JNK**

*E. coli* (10^7^ CFU/mL) - + - + + +

*L. plantarum* 17-5 (CFU/mL) - - 10^5^ 10^4^ 10^5^ 10^6^

46 kDa

60 kDa

44 kDa

35 kDa

45 kDa


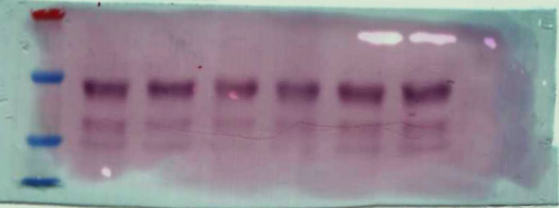


**JNK**

*E. coli* (10^7^ CFU/mL) - + - + + +

*L. plantarum* 17-5 (CFU/mL) - - 10^5^ 10^4^ 10^5^ 10^6^

60 kDa

46 kDa

45 kDa

44 kDa


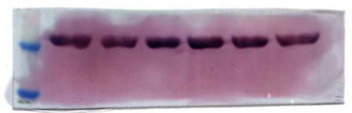


**β-actin**

*E. coli* (10^7^ CFU/mL) - + - + + +

*L. plantarum* 17-5 (CFU/mL) - - 10^5^ 10^4^ 10^5^ 10^6^

45 kDa

42 kDa

35 kDa
