# Supplementary material for: Lactobacillus plantarum 17–5 attenuates Escherichia coli-induced inflammatory responses via inhibiting the activation of the NF-κB and MAPK signalling pathways in bovine mammary epithelial cells
Source: BMC Vet Res. 2022 Jun 28;18:250. doi: 10.1186/s12917-022-03355-9 (PMC9238091; doi:10.1186/s12917-022-03355-9)

Additional file 2. Analysis of reference genes expression stability

The stability of *GAPDH* and five additional pairs of reference genes (*18S rRNA, ACTB, SDHA, UXT* and *YWHAZ*) were tested by GeNorm, NormFinder and BestKeeper. GeNorm, NormFinder and BestKeeper respectively showed the stability of the reference gene (Fig 1-3, this page). The comprehensive stability ranking order (Better--Good--Average) was *GAPDH*, *SDHA*, *UXT*, *18S*, *ACTB* and *YWHAZ* (Fig 4, this page).


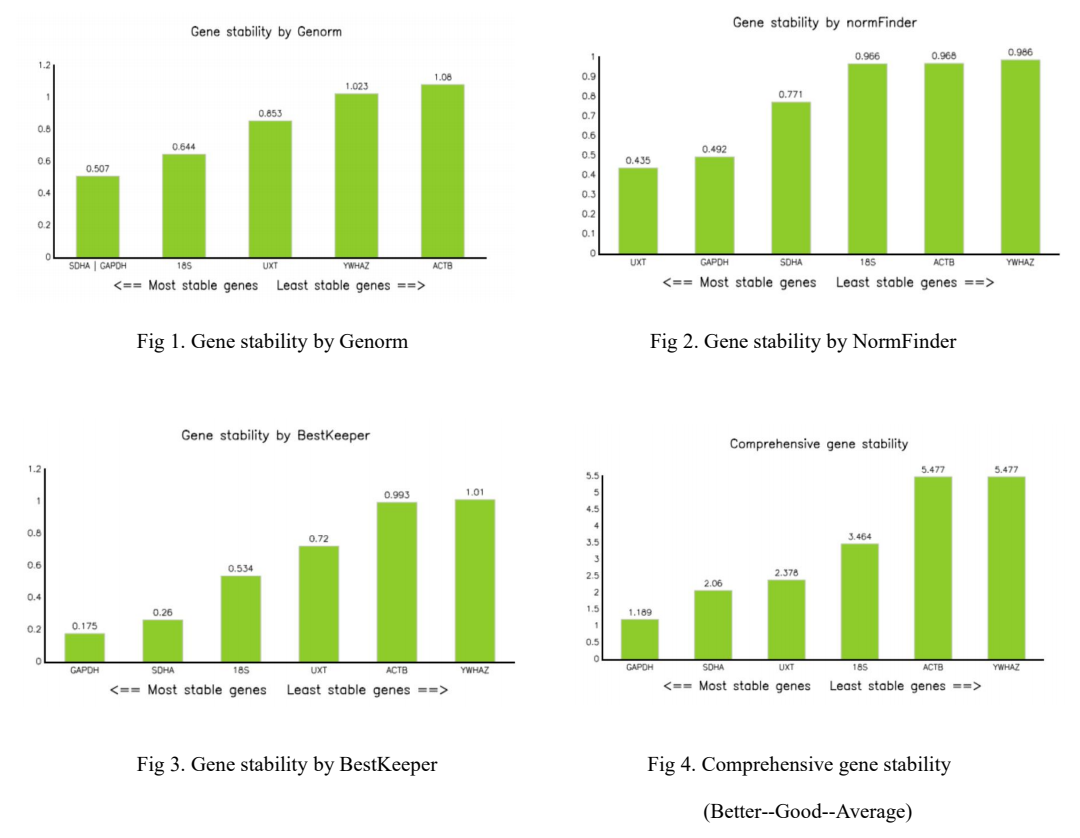

Supplement: Supplementary file 2 — Additional file 2. Analysis of reference genes expression stability. [file 12917_2022_3355_MOESM2_ESM.doc]
